# Supplementary material for: Conventional measures of intrinsic excitability are poor estimators of neuronal activity under realistic synaptic inputs
Source: PLoS Comput Biol. 2021 Sep 16;17(9):e1009378. doi: 10.1371/journal.pcbi.1009378 (PMC8478185; doi:10.1371/journal.pcbi.1009378)
Supplement: S1 Table — gsx is the electrical coupling conductance between the soma and axon compartments. gsd indicates the coupling between the soma and dendrite. (DOCX) [file pcbi.1009378.s001.docx]

|  | Soma | | Axon | | Dendrite | |  |  |  |
| --- | --- | --- | --- | --- | --- | --- | --- | --- | --- |
|  | *g_leak_*  nS | *C_m_*  pF | *g_leak_*  nS | *C_m_*  pF | *g_leak_*  nS | *C_m_*  pF | *g_sx_*  nS | *g_sd_*  nS | *E_leak_*  mV |
| Regular | 2.3 | 50 | 1.4 | 12 | 1.4 | 15 | 16 | 16 | -68.0 |
| Delayed | 2.0 | 65 | 1.4 | 20 | 1.4 | 20 | 16 | 16 | -64.0 |
| Stuttering | 2.6 | 40 | 1.6 | 12 | 1.6 | 15 | 16 | 16 | -66.0 |

**S1 Table.**
