# Supplementary material for: Conventional measures of intrinsic excitability are poor estimators of neuronal activity under realistic synaptic inputs
Source: PLoS Comput Biol. 2021 Sep 16;17(9):e1009378. doi: 10.1371/journal.pcbi.1009378 (PMC8478185; doi:10.1371/journal.pcbi.1009378)
Supplement: S2 Table — So, Ax and De indicate the percentage of conductance allocated for the somatic, axonic and dendritic compartments. (DOCX) [file pcbi.1009378.s002.docx]

| Curr | Typ | *g* | *E* | *So* | *Ax* | *De* | *p* | *V_m,1/2_* | *V_m,sl_* | *V_h,1/2_* | *V_h,sl_* | *τ_m,max_* | *τ_m,min_* | *V_tm,1/2_* | *V_tm,sl_* | *τ_h,max_* | *τ_h,min_* | *V_th,1/2_* | *V_th,sl_* |
| --- | --- | --- | --- | --- | --- | --- | --- | --- | --- | --- | --- | --- | --- | --- | --- | --- | --- | --- | --- |
|  |  | nS | mV | % | % | % |  | mV | mV | mV | mV | ms | ms | mV | mV | ms | ms | mV | mV |
| Na | R  D  S | 11000  14000  13000 | 55 | 25 | 75 | 0 | 3 | -26 | 14 | -56 | -14 | 0.9 | 0.1 | -68 | 30 | 8 | 0.5 | -74 | 30 |
| Na_P_ | R  D  S | -  -  1.0 | 50 | 33 | 0 | 67 | 1 | -26 | 13 |  |  | 3.0 | 0.2 | -70 | 50 |  |  |  |  |
| H | R  D  S | 3.0  -  4.0 | -40 | 25 | 0 | 75 | 1 | -73 | -16 |  |  | 200 | 15.0 | -62 | 30 |  |  |  |  |
| K_d_ | R  D  S | 220  320  200 | -78 | 25 | 75 | 0 | 4 | -24 | 15 |  |  | 10 | 0.7 | -70 | 30 |  |  |  |  |
| M | R  D  S | 4.0  20.0  5.0 | -78 | 50 | 50 | 0 | 1 | -28 | 17 |  |  | 80 | 20.0 | -80 | 100 |  |  |  |  |
| D | R  D  S | 40  100  600 | -78 | 50 | 50 | 0 | 3 | -36 | 15 | -60 | -8 | 10 | 1 | -80 | 70 | 180 | 100 | -90 | 70 |
| K_ir_ | R  D  S | -  15.0  - | -78 | 67 | 0 | 33 | 1 | -80 | -15 |  |  | 50 | 3.0 | -45 | 40 |  |  |  |  |
| Ca_T_ | R  D  S | 20  -  - | 90 | 100 | 0 | 0 | 2 | -49 | 14 | -83 | -13 | 5.0 | 0.8 | -68 | 30 | 50 | 5 | -75 | 25 |
| Ca_L_ | R  D  S | 10  18  5 | 90 | 50 | 0 | 50 | 2 | -23 | 13 | -53 | -13 | 10 | 1.0 | -54 | 25 | 50 | 3 | -65 | 25 |

**S2 Table.**
