# Supplementary material for: Conventional measures of intrinsic excitability are poor estimators of neuronal activity under realistic synaptic inputs
Source: PLoS Comput Biol. 2021 Sep 16;17(9):e1009378. doi: 10.1371/journal.pcbi.1009378 (PMC8478185; doi:10.1371/journal.pcbi.1009378)
Supplement: S3 Table — Maximal conductances of voltage-gated currents, membrane capacitance, leakage reversal potential, and coupling conductances between compartments were varied in a Gaussian distribution. The values are expressed as mean ± S.D. of the distributions. (DOCX) [file pcbi.1009378.s003.docx]

| Regular | Delayed | Stuttering |
| --- | --- | --- |
| H: 3.0 ± 1.5 nS  M: 4.0 ± 2.0 nS  CaT: 20.0 ± 10.0 nS  D: 40.0 ± 20.0 nS  CaL: 10.0 ± 5.0 nS  K(Ca): 50.0 ± 25.0 nS  Cm: 60.0 ± 12.0 pF  Eleak: -68.0 ± 2.0 mV  GSomAx: 16.0 ± 1.6 nS  GSomPr: 16.0 ± 1.6 nS | M: 10.0 ± 5.0 nS  D: 30.0 ± 15.0 nS  Kir: 10.0 ± 5.0 nS  CaL: 14.0 ± 7.0 nS  K(Ca): 50.0 ± 25.0 nS  Cm: 70.0 ± 14.0 pF  Eleak: -68 ± 2 mV  GSomAx: 16.0 ± 1.6 nS  GSomPr: 16.0 ± 1.6 nS | H: 4.0 ± 2.0 nS  M: 7.0 ± 3.5 nS  D: 1000 ± 500 nS  NaP: 1.0 ± 0.5 nS  CaL: 5.0 ± 2.5 nS  Cm: 40.0 ± 8.0 pF  Eleak: -67 ± 2 mV  GSomAx: 16.0 ± 1.6 nS  GSomPr: 16.0 ± 1.6 nS |

**S3 Table.**
