# Supplementary material for: Conventional measures of intrinsic excitability are poor estimators of neuronal activity under realistic synaptic inputs
Source: PLoS Comput Biol. 2021 Sep 16;17(9):e1009378. doi: 10.1371/journal.pcbi.1009378 (PMC8478185; doi:10.1371/journal.pcbi.1009378)
Supplement: Attachment — Submitted filename: Response to Reviewers 21721.docx [file pcbi.1009378.s006.docx]

Response to the Reviewers

Manuscript: " Conventional Measures of Intrinsic Excitability Are Poor Estimators of Neuronal Activity Under Realistic Synaptic Inputs " by Adrienn Szabó et al. (manuscript number: **PCOMPBIOL-D-21-00383**)

The authors thank the Reviewers for their careful reading of the manuscript, their expert suggestions and supportive comments. We made an effort to revise the manuscript according to the Reviewers’ suggestions, by adding new data that further support our findings and by expanding the Discussion. Our point-by-point response to the Reviewers’ suggestions are listed below.

Reviewer #1: The authors conducted a thorough analysis of the roles of Kv1 and Kir K+ currents in excitability of neurons by comparing electrical responses of cultured mouse hippocampal neurons to standard long square current protocols and to injected fast artificial synaptic inputs via dynamic clamp. They found that there are at least three types of neurons separated by their response to long pulses of current. Two of these types exhibited opposite effects of these stimulation protocols. They found that the responses of neurons classified as delayed firing type, could be explained by relatively upregulated KIR current; and the responses of neurons described as being of stuttering type are explained by relative upregulation of Kv1 channels (D-type K current). These study cleverly compares results of electrophysiological measurements, pharmacological blockade of the key currents, dynamic clamp stimulation, and computational modeling. The study is clearly written and provides interesting and general results. If questions and issues described below are properly addressed this study could have high impact in the field of neuroscience.

We thank the Reviewer acknowledging the merit of our study and for the expert suggestions he/she made.

Major

I suggest that these classifications and types of responses should also acknowledge the role played by hyperpolarization activated (h-) current in the stuttering neurons. Figure 1 B suggests that neurons with the delayed firing practically have no h-current while stuttering neurons and regular firing neurons have prominent sag potential reporting notable presence of h-current. In the model this distinction is less obvious.

Indeed, the Reviewer is right recognizing that both regular firing and stuttering type neurons express the h-current and display prominent voltage sag in their static responses – we thank him/her for raising this point.

In fact, regular and stuttering type neurons are similar in this respect and differ mainly how strong outward rectification they produce. As our computational modeling and experiments with Kv1-channel blockers demonstrated, outward rectification is primarily caused by the D-current that is strong in stuttering neurons and weaker or undetected in regular type cells. We extended our manuscript to discuss these differences and their potential consequences. Inspecting the R_max,0_ vs. V_rest_ scatter plots in Fig. 1J we notice that stuttering type neurons tend to have lower membrane resistance than regular firing cells. This is mainly the result of their potent intrinsic h-current that acts as shunting at resting V_m_ and at more hyperpolarized potentials. However, this strong h-current affects intrinsic excitability and synaptic integration in a way that is somewhat convoluted. On the one hand, shunting decreases static firing responses and the accumulation of excitatory synaptic potentials, therefore it has a negative effect on excitability. On the other hand, the resting membrane potential of such neurons is more depolarized than those without h-current. We have previously investigated the effects of h-current block in hippocampal neurons (Rátkai et al. 2021, Sci. Reports) and observed a clear hyperpolarizing shift of 5-6 mV magnitude after the application of ZD7288, a specific antagonist of the h-current. Indeed, the h-current is partially activated at resting membrane potentials, therefore it pulls V_m_ toward -39 mV, which is the estimated reversal potential of this current in cultured hippocampal neurons. Due to I_h_, the resting membrane potential shifts toward the voltage threshold of action potentials, hence, neurons reach firing threshold easier under excitatory inputs or current stimulation. Altogether, we find that the effect of I_h_ on intrinsic excitability is more complex than that of I_Kir_ or I_D_. Lines 534-555 are added to Discussion to address these.

Regarding the model neurons, we included the Ih in both the regular and the stuttering phenotypes but not in the delayed type. Stuttering model neurons, therefore, also exhibit the voltage sag as regular firing ones.

It appears that the role of h-current contributed to a counter-intuitive change in responses after blockade of Kv1 channels. “Somewhat paradoxically, the latency parameter increased after D-current removal in the stuttering models (Fig. 5D) indicating that these cells start firing with longer

latencies but at lower step current levels compared to those having their intrinsic D currents intact.” Could this interesting phenomenon be explained by partial deactivation of h-current? This could be easily tested in the model and would serve as strong validation of the model and shed light on the role of h-current.

We thank the Reviewer for this observation and we are glad to provide clarification for these findings. We investigated the role of the h-current in the stuttering type neurons by performing model simulations and the results from such computations are now presented in the supplementary figure S1. We compared the latency, membrane resistance and cumulative spike count parameters of reduced stuttering model neurons (without I_h_) in settings identical to that in Fig. 5. Essentially, we found a minor effect of I_h_ in regulating firing responses of the model neurons and we added a new section to discuss these results (lines 534-555).

Left: Model neuron exhibiting slow ramp to the first spike and discontinuity in the latency curve. Here, the latency function starts near 400 ms (y-axis) and drops markedly when the initial voltage bump at the onset of the stimulus (top trace, t=0.02 s) becomes a full action potential. Right: Model neuron starting the firing response with a single spike and producing a smooth, continuous latency function that descends from 22.5 ms.

Traditionally, the D-current mediated by Kv1 channels is associated with its effect on prolonging spike latency under depolarizing current step injection. Indeed, most reports on the functional effects of the D-current demonstrate such action and the pharmacological removal of this current decreases the latency to the first spike. The demonstration of the spike delaying effect of the D-current is usually done by showing a single trace of firing response before and after the application of dendrotoxin or 4-AP. However, we notice that latency is better investigated as a function of the injected current and we prefer comparing these latency functions obtained from different neurons or when applying pharmacological manipulation. In fact, first spike latency appears to be a clean, monotonically decreasing function of the current. Apart from the first 1 or 2 datapoints close to rheobase, the entire latency function can be well fitted with a Belehradek-function of the form Lat = a*(*I*-b)^c^, where I is the current. We are using these functions to obtain a smooth mathematical representation of the spike latency across different neurons and in a way that takes into account behavior close to rheobase and at more depolarized levels, too. Clearly, pharmacological blocking of a membrane current will impact the entire latency curve by shifting individual latency values, so it is not easy to characterize such effects using a single metric. On the other hand, we found that spike latency at a current level slightly over rheobase is informative and can be used to compare a wide variety of neurons and effects of biophysical manipulations. This parameter is the spike latency at a current level 30% higher than the rheobase of the neuron (Latency_1.3_), it is sensitive to blocking/removal of specific currents and it is more reliable/accurate than the latency observed very close to the rheobase.

At the same time, the well-known effect of the D-current is much better demonstrated near rheobase, where spike latencies tend to be very long. Also, the well-known effect of this current is not always readily found when neurons tend to emit single spikes in response to currents slightly above rheobase. In fact, most of our biological neurons from the hippocampal culture exhibit this type of behavior rather than a slowly rising voltage ramp to the first spike. Our model neurons well reproduced this behavior, and, depending on the magnitude and kinetics of the D-current, we observed both single spiking and delayed firing in such model cells. It is important that single spiking type model cells and also the same type of biological neurons require a strong depolarizing current to initiate their firing. Also, when the current becomes even higher, they start to emit multiple spikes but in an irregular pattern, effectively this is when they are stuttering. So, their rheobase is high, but their first spike is emitted at the beginning of the pulse. When the intrinsic D-current is blocked in such neurons, their rheobase drops very significantly, and their behavior becomes more similar to that of the regular firing cells. Their latency curve moves to the left along the current axis. At the same time the latency functions of such “regular firing” cells (without D-current) descend from higher values than those of the single spiking cells (strong D-current). This is why the paradoxical effect of D-current removal appears, i.e. the first spike latency at 1.3-times of the rheobase becomes prolonged.

Considering all these, we believe that the characteristic behavior seen in the stuttering/single spiking cells is mainly determined by the action of the D-current and its strength is a major factor in setting the excitability of the neuron. In such cells, the intrinsic h-current has less impact in shaping the firing under depolarizing current pulses and the latency of the first spikes is also relatively insensitive to the magnitude of I_h_. Our supplementary figure S1 demonstrates this point. While the membrane resistance of the model neurons is clearly increased by removing the h-current, first spike latencies are barely affected by this manipulation.

These two types of neurons appear to represent type I and type III excitability. It would help this study to discuss presented results in the context of types of excitability.

We agree with the Reviewer regarding the justification of discussing the firing responses in the context of excitability types. We extended our manuscript to discuss the connection between firing modes and excitability types (lines 476-493). The Zeberg paper we refer to is especially relevant, because the authors use dynamic clamp to investigate biophysical mechanisms facilitating type 2 excitability and stuttering. Their results are, in many respects, in agreement with our observations.

The applicability of the term stuttering is not obvious for me and should be explained and well defined as a set of clear formal criteria for classification. It appears that the authors determine this type of neurons by the predominantly silent interval after initial response of neurons to long square current pulse. Are these neurons bursting? How many spikes are in the groups of spikes? Two? Are there more spikes in the first bursts/groups? Could these bursts be due to reminiscent activated h-current?

In the revision we described in more details what criteria we used to categorize neurons as stuttering type (lines 137-140). The Reviewer is right that our primary criterion was to observe one or more silent intervals during the depolarizing current step, i.e. intermittent firing. In effect, these silent intervals separate the firing response into packets or bursts of action potentials. However, we refrain from using the term burst here. We find no indication of Ca-spikes in such neurons where fast action potentials appear on top of slower depolarizing “bumps”. In the stuttering neurons episodes/packets of regular firing can be separated by longer silent intervals when characteristic subthreshold oscillations can be seen. Within the spike packets we can observe variable number of spikes ranging from 2 to 6. True bursters with strong Ca-currents tend to emit spikes with variable interspike intervals (ISI envelope), and the membrane potential between bursts exhibits more pronounced minima. Another important feature that helps identifying the stuttering neurons is the marked spike afterhyperpolarization. Action potentials are sharp and there is a deep repolarization when the membrane potential falls more than 10 mV below the voltage threshold of the spike. This spike AHP can therefore be used as a quantitative measure that separates stuttering neurons from the regular or delayed firing types. As one more interesting feature, stuttering neurons often emit a single action potential in response to current injections slightly above rheobase. Later, a fairly periodic firing pattern can be seen, hence, single spikes are followed by multiple ones causing a big jump in the I-O function of the neuron. Indeed, discontinuity and irregularity of the spike count vs. current relationship is a hallmark of stuttering neurons and this is equally applicable to biological and model neurons. Altogether, stuttering, as used in our manuscript is a slightly broadly defined term, it refers to neurons that have higher rheobase than regular type cells, fire irregularly, exhibit discontinuous I-O function, produce and subthreshold oscillations under prolonged interspike intervals.

Relation of the results of this study to the “activity-dependent regulation of intrinsic excitability” should be better explained.

Our study did not directly address activity-dependent regulation of intrinsic excitability and our experiments were not designed to elicit such type of adaptations in neurons. The experiments most closely related to these phenomena were the ones where we applied specific antagonists of the D-current and I_Kir_. Such pharmacological manipulations mimic, at least partially, the neuroadaptive changes under LTP-IE, as an example, those in the Campanac et al. 2013 paper. Although entirely blocking a specific voltage-dependent current likely produces a stronger overall effect on firing output than activity-dependent downregulation, we believe, the comparison of our results to those from prior studies is still justifiable. In fact, authors commonly utilize pharmacological inhibition of voltage-gated currents to reproduce downregulation of the same currents after LTP-induction. Physiological parameters of hippocampal neurons with different magnitude of D-current tend to “move” in a consistent manner after the application of the blocker and we find the same for the model neurons after removal of the D-current. Considering all these we believe, the biophysical manipulations we carried out are useful to understand and predict functional changes under activity-dependent regulation of intrinsic properties. We extended the Discussion to emphasize these points (lines 446-455).

Minor

Figure 1 A, B, and C should have same voltage scale bar.

Corrected.

Are neurons with rest membrane potentials above -50 mV excitability blocked?

Cultured hippocampal neurons 12-14 days after plating tend to be slightly more depolarized than similar type of neurons in acute brain slice preparations from juvenile or adult animals. We are aware of this difference and we think the excitability (I-O functions) of these neurons might also be influenced by the depolarized resting V_m_. However, we found no clear signs of depolarization block under current step stimulation or when using simulated synaptic bombardment. In general, depolarization block has a strong impact on spike amplitudes and the neuron’s ability to maintain rapid firing under long current pulses. However, in our experiments, spike amplitudes were rarely below 60 mV and healthy firing responses were recorded even when the resting V_m_ was above -50 mV. We believe, the comparison of static vs. dynamic firing responses of neurons including the ones with relatively more depolarized resting V_m_ is justified, because the main findings of our study are equally applicable to neurons with -65 and -50 mV resting V_m_. Also, neurons with depolarized resting V_m_ require less current to fire (lower rheobase), but they exhibit a monotonously growing input-output relationship. Stuttering neurons are, of course, are different in this respect, because their firing rate is less correlated with the magnitude of the current injection, but they can still produce sharp spikes at current levels 2-times that of their rheobase.

Here and below, the pictogram of experimental recording in the top right corner is blurred and not contrast. It should be improved.

Corrected as requested.

186: “threshold AMPA-conductance as a function of the threshold current level (rheobase) for” the term rheobase could be introduced as the threshold current level earlier in the text.

We corrected this mistake and defined the term in the revised manuscript as requested (line 122).

Experimental rest potentials are much more depolarized than model rest potentials. Could the authors discuss this discrepancy.

We agree that the resting membrane potentials of cultured neurons are significantly higher than those of the model neurons. Model neurons better represent biological neurons that exist in established, mature brain circuits such as in hippocampal slice preparations. The depolarized resting V_m_ of cultured neurons are probably due to their relative immaturity, i.e. because they are still less differentiated than neurons in the brain tissue. However, we find no adverse effects of the relatively depolarized resting V_m_ when performing electrophysiological experiments in such cultured cells. Whole cell and dynamic clamp recordings of duration up to 20 minutes can be routinely performed with stable firing responses and physiological parameters.

Figure 3C it is not clear whether the neurons spiking at the end of stimulation produced spikes in the beginning. Probably, if the traces are plotted in different colors then the traces could be distinguished.

We agree with the Reviewer’s suggestion, because it is, indeed, difficult to see how the firing of the stuttering type neuron evolved as the injected current was increased. As in many, but not all model examples, the first action potential appeared at the beginning of the step and spike followed after a prolonged silent interval at more depolarized levels. This model exhibits a clear example of discontinuity in the I-O function, a signature of stuttering type neurons. Also, this behavior is similar to that of type 2 excitability, although the firing rate rarely jumps from zero to a high level, instead, a single spike is emitted under 2-5 consecutive current steps and then it jumps to higher counts (Fig. 1H, 2F, 4H). We tried several arrangements of the traces to show how the firing developed with increasing current level. Eventually, we decided to depict the first spike response for each phenotype as a single separated trace, because this seemed to be the best solution. We have now blue voltage traces appearing in Fig. 1 and 3.

Figure 3I does not show data and thus it could not match experimental data.

We apologize for this mistake. This problem occurred because of the conversion of the Word document into PDF. The panel 3I in our Word document (DOCX) was correct and all the data appeared as intended, however, points disappeared in the PDF document. The problem seems to persist and data are still not displayed in the PDF for unknown reasons. We include this panel here.

Figure 3 J visibly exhibits a trend different from experimental.

Indeed, comparing Fig 1J and 3J for the biological vs. model neurons we recognize that the latter ones cannot fully reproduce the variability of experimental data. We believe this is probably due to the limitations of our model. It is likely that including additional variations in our model neurons such as the spatial distribution (allocation) of voltage-gated currents or kinetic parameters of those would further improve the qualitative match between experimental observations and the model data. We will explore this possibility in future simulations.

227 “For each model phenotype we generated 200 instances and subjected those to the two types of stimulation as used with the biological neurons.“ How did you produce these 200 instances? Did the model phenotype appear as an emergent property? If not, what constraints did you use to obtain them?

Maximal conductances of the voltage-dependent membrane currents were varied randomly using a Gaussian distribution. We added a supplementary table (S3) to provide the exact ranges for the varied parameters. Following the selection of maximal conductance parameters, the model implementations were subjected to the current step stimulation and simulated synaptic bombardment as described. The model phenotypes were based on our prior computational studies that were designed to investigate differential regulation of firing output of model neurons as well as cultured hippocampal neurons (Szücs and Huerta, 2015; Szücs et al., 2017).

233 Is the model multicompartmental?

The model consisted of a somatic, a dendritic and an axonal compartment. Current step stimulation was applied on the somatic compartment while simulated synaptic inputs were targeting the dendritic compartment.

What would 4ap application do to the delayed neurons? What would application of BaCl2 do to stuttering neurons? The data should be shown:” It is also noteworthy that the actions of the two channel blockers were cell type specific, meaning that BaCl2 was found to be ineffective when applied on stuttering type neurons while 4-AP was ineffective on delayed firing type ones (data not shown). These observations indicate that Kir and Kv1 channels are expressed in separate populations of cultured hippocampal neurons.”

We carefully checked our data and found that our original phrasing was probably too strict. In fact, we found examples when BaCl_2_ was indeed ineffective on the overall firing output, voltage sag, membrane resistance and time constant of stuttering neurons, clearly indicating that the K_ir_-current was likely not expressed in such type of neurons. On the other hand, application of 4-AP did not change the inward rectification, membrane resistance and spike latency curve of delayed firing type neurons, but their cumulative spike count was still increased by this manipulation. This can be a consequence of a lower magnitude D-current present in some delayed firing type neurons. In fact, we did include the D-current at 30 ± 15 nS conductance level in the 200 implementations of delayed firing type model neurons. Hence, we prefer to remove the above mentioned sentences.

Our reasoning on the use of pharmacological blockers, we believe, is still justified and the results can be correctly interpreted, because BaCl_2_ and 4-AP targets different populations of voltage-gated channels and such actions can be well reproduced in computational models, too. Also, these two drugs were always used separately, not in combination, on different types of neurons. 4-AP specifically and reliably blocked the D-type K-current in stuttering neurons, while BaCl_2_ blocked the K_ir_-current in delayed type neurons. In this respect, the action of a weaker D-current in delayed firing type neurons was not relevant, because BaCl_2_ had no effect on that. It is also noteworthy that the characteristic voltage ramp observed in delayed firing type neurons is a direct consequence of the deactivation of the K_ir_-current and not regulated by the D-current in such neurons. BaCl_2_ reliably shortened the spike latency in delayed type neurons.

Why figure 4 is not reproduced in the model?

Figure 5 is actually analogous to that of Fig. 4 but showing physiological parameters (Fig 5A, B) instead of representative voltage responses (Fig. 4A, B, H, I) and input-output curves (Fig. 4C, D, J, K). Total spike counts after the blocking/removal of the Kir-current and the D-current are shown for both biological and model neurons.

Why model analysis in Figure 5 does not have a corresponding experimental figure?

In the model simulations we were able to test a large number of model neurons and the effects of removal of selected voltage-gated currents. 200 model implementations were sufficient to demonstrate the differential effects on firing responses. Biological experiments were far more challenging in the technical sense, because we aimed for the complete physiological characterization of neurons, the acquisition of their spike responses under static and dynamic inputs and to measure the effects of blockers of voltage-dependent currents, too. It was not feasible to construct densely populated 2D scatter plots of physiological parameters such as the ones created for the model data (Fig. 5A, B, D, E). Still, we believe, the most important points are well demonstrated: static vs. dynamic firing responses are differentially regulated by BaCl_2_ and 4-AP in the two types of hippocampal neurons. Panels in Fig. 4E, F, L, M have their corresponding panels in Fig. 5C, F and there is a good agreement between experimental and model data.

Reviewer #2: This manuscript combines electrophysiology and dynamic clamp experiments on cultured rat hippocampal neurons with computational ensemble modeling. The major conclusion drawn by the authors is that the voltage responses elicited from neurons by traditional current step stimuli are a poor predictor of the effect of more realistic stimuli (in the form of barrages of excitatory and inhibitory synaptic inputs) on neuronal firing rates and other excitability measures. Two potassium membrane currents are identified as major determinants of physiologically meaningful excitability measures. Interestingly, the effects of these membrane currents can be in the opposite direction for realistic stimulation compared to step current stimulation, and can differ between neuron types (the authors distinguish three types based on the temporal spiking profile and the presence or absence of sag currents).

The experiments and models are well thought out and executed. In particular, the use of ensemble modeling to replicate biological variability across populations of neurons is highly appropriate. The conclusions are well founded in the experimental and modeling results, the figures are clear, and the text is concise and to the point.

I have only one comment that I hope the authors can address: the literature cited could more thoroughly relate the results presented here to previous work in the literature. Most notably, it is surprising that the seminal work of Mainen and Sejnowski on the poor suitability of current step stimuli for the characterization of neocortical response dynamics is neither cited nor discussed here: Mainen ZF & Sejnowski TJ (1995). Science 268: 1503-1505.

We thank the Reviewer for the kind and positive remarks and his/her suggestion to discuss the applicability of dynamic clamp to investigate neuronal response properties. We fully agree with the Reviewer that temporally complex synthetic synaptic inputs that mimic in vivo activity are well suited to probe cellular responses as well as circuit interactions. In fact, some of our earlier reports focused on spike timing reliability and precision on biophysically different type of neurons (Szücs et al. 2004, 2010). We are therefore happy to extend the Discussion to highlight some of the findings investigators revealed using this technology (lines 598-607). While our primary focus in this study was the regulation of intrinsic excitability and firing rate, we are well aware of the fact that neurons exhibit sophisticated, precisely timed spike responses under natural stimuli that require detailed investigations. We believe, a more lengthy discussion including how spike timing reliability and precision is regulated by the spectral properties of the synaptic input and the preceding membrane potential history of the postsynaptic neuron would be worthy. Yet, we hope the Reviewer will agree that briefly mentioning these directions is suitable within the scope of our present study.
